# The World Health Organization was born as a normative agency: Seventy-five years of global health law under WHO governance

**DOI:** 10.1371/journal.pgph.0002928

**Published:** 2024-04-11

**Authors:** Lawrence O. Gostin, Benjamin Mason Meier, Safura Abdool Karim, Judith Bueno de Mesquita, Gian Luca Burci, Danwood Chirwa, Alexandra Finch, Eric A. Friedman, Roojin Habibi, Sam Halabi, Tsung-Ling Lee, Brigit Toebes, Pedro Villarreal

**Affiliations:** 1 O’Neill Institute for National & Global Health Law, Georgetown Law School, Washington, District of Columbia, United States of America; 2 Gillings School of Global Public Health, University of North Carolina at Chapel Hill, Chapel Hill, North Carolina, United States of America; 3 Berman Institute of Bioethics, Johns Hopkins University, Baltimore, Maryland, United States of America; 4 Human Rights Centre, Essex Law School, Colchester, United Kingdom; 5 Global Health Centre, Graduate Institute of International and Development Studies, Geneva, Switzerland; 6 Faculty of Law, University of Cape Town, Cape Town, South Africa; 7 Faculty of Law, University of Ottawa, Ottawa, Canada; 8 Graduate Institute of Health and Biotechnology Law, Taipei Medical University, Taipei, Taiwan; 9 Faculty of Law, University of Groningen, Groningen, The Netherlands; 10 Max Planck Institute for Comparative Public Law and International Law, Heidelberg, Germany; University of Ottawa, CANADA

## Abstract

The World Health Organization (WHO) was born as a normative agency and has looked to global health law to structure collective action to realize global health with justice. Framed by its constitutional authority to act as the directing and coordinating authority on international health, WHO has long been seen as the central actor in the development and implementation of global health law. However, WHO has faced challenges in advancing law to prevent disease and promote health over the past 75 years, with global health law constrained by new health actors, shifting normative frameworks, and soft law diplomacy. These challenges were exacerbated amid the COVID-19 pandemic, as states neglected international legal commitments in national health responses. Yet, global health law reforms are now underway to strengthen WHO governance, signaling a return to lawmaking for global health. Looking back on WHO’s 75^th^ anniversary, this article examines the central importance of global health law under WHO governance, reviewing the past successes, missed opportunities, and future hopes for WHO. For WHO to meet its constitutional authority to become the normative agency it was born to be, we offer five proposals to reestablish a WHO fit for purpose: normative instruments, equity and human rights mainstreaming, sustainable financing, One Health, and good governance. Drawing from past struggles, these reforms will require further efforts to revitalize hard law authorities in global health, strengthen WHO leadership across the global governance landscape, uphold equity and rights at the center of global health law, and expand negotiations in global health diplomacy.

The World Health Organization (WHO) was born as a normative agency with unrivaled constitutional authority to negotiate and adopt international legal instruments to advance global health governance. Yet, it has adopted only two major legally binding instruments in its history—the International Health Regulations (IHR) and the Framework Convention on Tobacco Control (FCTC). In the wake of the COVID-19 pandemic, WHO is embarking on a fundamental reform of the IHR and is negotiating a new Pandemic Agreement. As the Organization looks back on the past 75 years and looks ahead to meet rising health challenges, WHO must harness its legal authorities to shape global health rules and norms and facilitate accountability for them–strengthening global health law to become the normative agency it was meant to be.

Applying public international law to address global health challenges, global health law looks beyond the legal efforts of individual nations to encompass the larger set of determinants that structure public health in a globalizing world. Global health law encompasses both binding “hard” law treaties and non-binding “soft” law instruments that shape norms, processes, and institutions to realize the highest attainable standard of physical and mental health. WHO holds expansive normative authorities to develop global health law instruments, including conventions, agreements, regulations, recommendations, standards, and nomenclatures. As contrasted with the agency’s technical or operational functions, these international legal instruments provide a powerful tool to meet major global health challenges that no country could address on its own. Recognizing that all nations face interconnected public health threats, global health law can foster collective action to realize global health with justice under WHO governance.

However, WHO has faced legal challenges throughout its 75-year history, revealing the limits of global health law as a foundation of global health governance. WHO has long been challenged by state assertions of national sovereignty, with member states often reluctant to establish binding obligations and at times failing to abide by existing obligations. During the COVID-19 pandemic, WHO confronted unprecedented legal challenges, with states neglecting international legal commitments in the pursuit of nationalist health responses. These constraints on WHO governance undermined global solidarity, leading member states to initiate sweeping law reforms to meet future health threats.

Where WHO leadership has been shaped by global health law, law reforms under WHO’s normative authorities will be essential to strengthen WHO governance. Given the limitations of global health law–in the COVID-19 response and beyond–it will be crucial for law reforms to take account of lessons learned from past challenges to ensure that WHO is prepared for future threats. This review examines the central importance of global health law under WHO governance, providing necessary understanding of the past successes, missed opportunities, and future hopes of legal authorities for global health.

## Legal authorities for global health

The normative foundations of the modern field of global health law were established by the WHO Constitution [1]. Declaring “the highest attainable standard of health” to be “one of the fundamental rights of every human being,” the WHO Constitution endowed WHO with a range of normative authorities to “act as the directing and coordinating authority on international health work” [2]. Upon its establishment in 1948, WHO set out to realize its international health mandate through global health law–leveraging its constitutional authorities to develop binding international legal agreements [3]. Yet despite its far-reaching lawmaking powers under the WHO Constitution, WHO has largely sought to advance global health governance through technical guidance, country support, and soft law policies, limiting accountability for international action [4].

The WHO Constitution grants WHO expansive international legal authorities to adopt international conventions, regulations, and non-binding recommendations to address matters of public health importance [5].

Establishing hard law authorities, Article 19 empowers the World Health Assembly to “adopt conventions or agreements” with respect to any matter within WHO’s competence. However, in its 75-year history, the Organization has adopted only one binding treaty–the FCTC. In addition to treaty powers, Article 21 empowers the Assembly to adopt binding regulations in enumerated areas of global health (international spread of disease, public health nomenclature, diagnostic procedures, and international trade of biological and pharmaceutical products). Under Article 22, these regulations are *automatically* binding on WHO member states unless a state explicitly opts out. Building from the long evolution of International Sanitary Conventions, first adopted in 19^th^ Century, WHO member states rapidly extended this normative authority to develop binding regulations through the adoption of the 1951 International Sanitary Regulations, which would be renamed in 1969 as the IHR, governing international action “to prevent, protect against, control and provide a public health response to the international spread of disease” [6].

**Fig 1:**
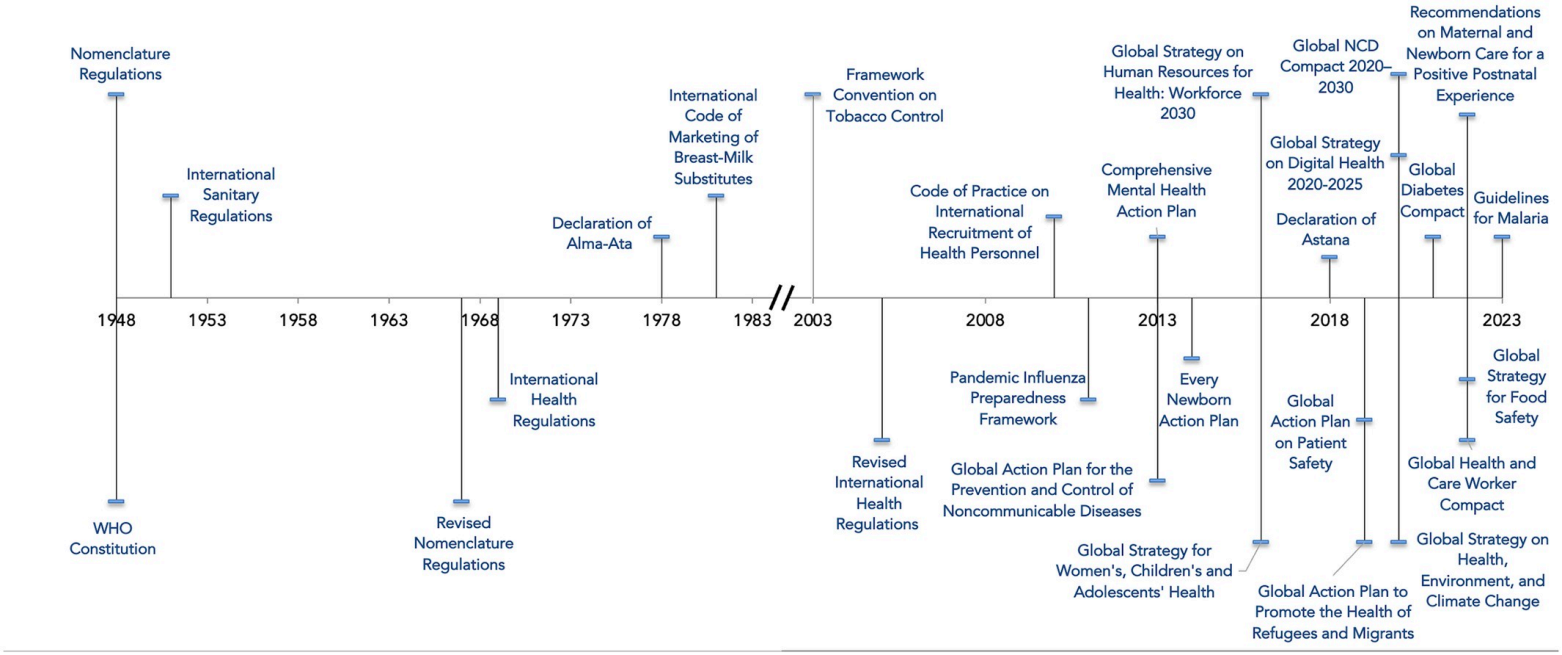
Select hard and soft law instruments under WHO governance.

Beyond hard law, WHO has turned to a wide range of soft law instruments, including resolutions, guidelines, action plans, global strategies, codes of practice, declarations, and recommendations. While these instruments do not codify binding obligations under international law, soft law can nonetheless be normatively authoritative [7]. Article 23 of the WHO Constitution empowers the Assembly to adopt nonbinding recommendations, which have established important global health norms, including on the marketing of breast-milk substitutes, the sharing of novel influenza viruses, and the recruitment of health care workers. Outside of formal recommendations adopted by member states, the WHO Secretariat has deployed an expanding series of soft law instruments to harmonize national health policies (Fig 1). These nonbinding instruments have addressed pressing health issues as broad as unhealthy diets, climate change, and digital health. The flexibility of soft law has allowed WHO to deploy these instruments rapidly and frequently to provide normative clarification of treaty obligations, offer technical guidance on health matters, set health standards for national governments and non-state actors, and ensure international accountability across an expanding global health landscape [8].

## New actors in an expanding global health landscape

**Fig 2:**
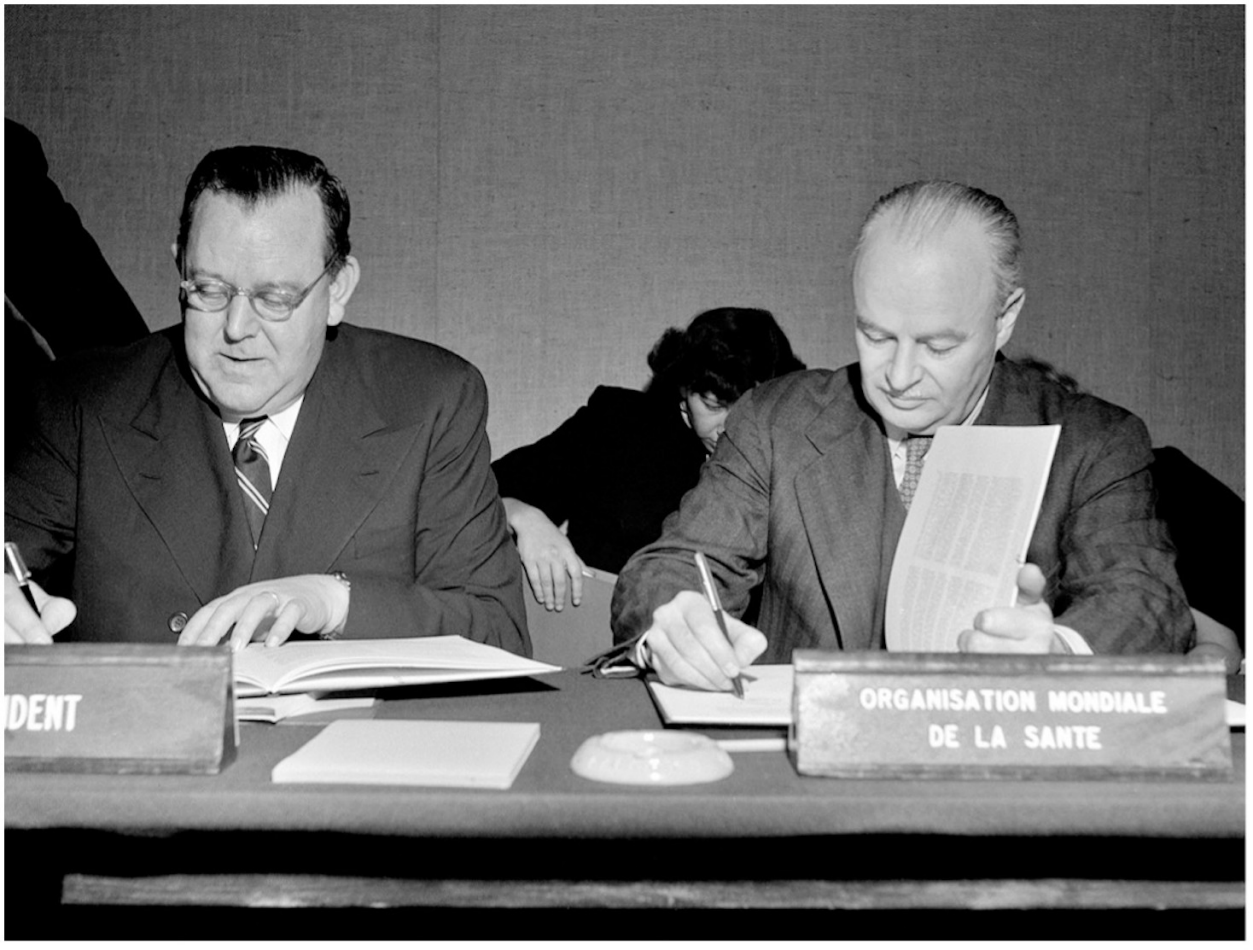
UN secretary-general Trygve lie and WHO director-general brock Chisholm formalize WHO becoming a UN specialized agency (Paris, November 1948). Republished from the World Health Organization under a CC BY license, with permission from the World Health Organization.

The establishment of the United Nations (UN) system after World War II provided the modern international legal foundation for global public health under WHO, as states looked to WHO to lead international legal advancements at the forefront of global health governance. As the principal UN specialized agency for health, WHO inherited from predecessor organizations the legal responsibility for multilateral cooperation to control the international spread of disease [9]. Yet, with its constitutional mandate to act as the “directing and coordinating authority” on all international health work, WHO would have far broader normative authority for international health lawmaking [10]. In coordinating the global community to address common health threats, WHO was central to global health governance under the UN, binding states together through international law to prevent disease and promote health (Fig 2).

However, amid political and economic shifts—from Cold War divisions to neoliberal economic challenges—WHO struggled to maintain its institutional leadership and normative influence [11]. The Cold War divided international relations into two opposing ideological camps—pitting Western capitalist democracies against Soviet communist regimes—with these geopolitical divides limiting WHO leadership to develop legal instruments [12]. Mounting tensions between the Soviet bloc and Western states rapidly undermined the solidarity needed within the World Health Assembly to develop hard and soft norms in global health–with Soviet states seeking to withdraw from WHO entirely for several years in the early 1950s [13]. Amid these geopolitical tensions, WHO avoided lawmaking initiatives at the height of the Cold War, with contemporaneous legal scholars noting that WHO had “regarded it a prudent tactic to rely less on regulations and more on the authority of international biomedical consensus,” recognizing that this “may not in all instances be as effective as a formal international health regulation” [14]. Even as Cold War tensions receded, high-income Western states continued to challenge WHO under a “neoliberal” approach to international development, shifting international health funding away from WHO and toward new vertical initiatives with more narrow mandates, giving rise to an expanding landscape of global health governance [15].

This expansion of the global health landscape at the end of the 20^th^ century fragmented WHO governance, weakening its institutional impact and sowing doubt in its international leadership [16]. Beginning in the HIV/AIDS pandemic, states saw WHO as inadequate to lead the global response, establishing the Joint United Nations Programme on HIV/AIDS (UNAIDS) to coordinate efforts across the UN. Without WHO’s singular leadership, a proliferation of new actors arose to address an array of health determinants–many joining together in global public-private partnerships with corporate actors, including the pharmaceutical industry [17]. The turn of the century saw the establishment of influential new global health partnerships, including Gavi, the Vaccine Alliance (2000) and the Global Fund for AIDS, Tuberculosis, and Malaria (2002). Many of these initiatives brought together a diverse range of national governments, philanthropic foundations, transnational corporations, and international organizations to implement disease-specific programming [18]. As WHO shifted from the unquestioned leader in international health to a more contested role amid these new actors in the global health landscape, the Organization has struggled to maintain its leadership in global governance to advance health as a human right.

## Human rights as a foundation of health justice

The human rights foundations of global health law were laid by the 1945 UN Charter, which affirmed human rights as a core pillar of the UN system, and the 1946 WHO Constitution, which proclaimed health as a fundamental human right. The 1948 Universal Declaration on Human Rights became the fundamental UN proclamation of human rights, but it would take nearly two decades to translate this initial UN declaration into binding legal obligations–under the 1966 International Covenant on Civil and Political Rights and International Covenant on Economic, Social and Cultural Rights, with the latter treaty enshrining the right to the “highest attainable standard” of physical and mental health [19]. This right to health would become central to political advocacy for WHO policies, programs, and practices, yet the Organization long struggled to mainstream human rights across its governance structures [20].

**Fig 3:**
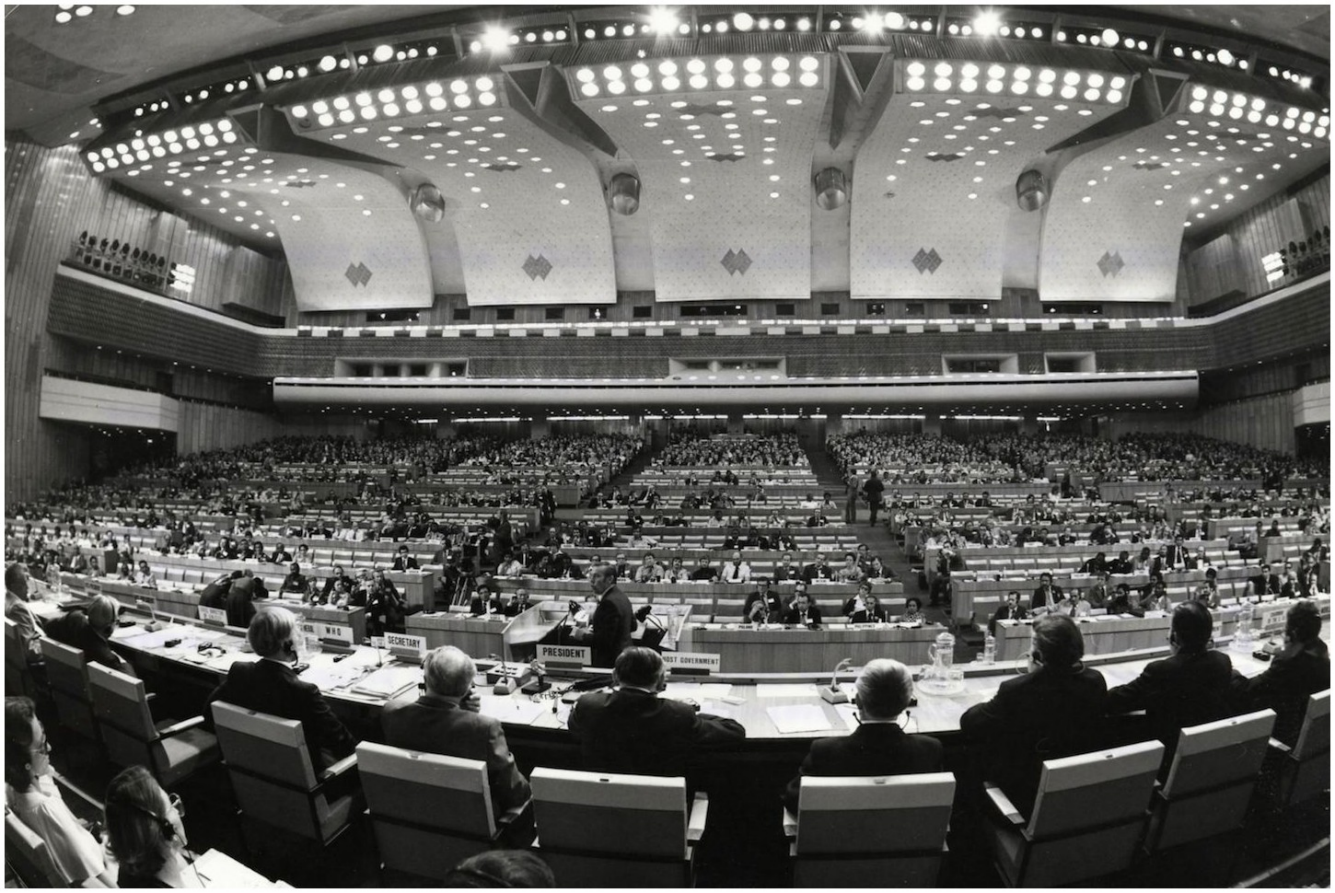
WHO director-general Halfdan Mahler addresses the international conference on primary health care (Alma-Ata, September 1978). Republished from the World Health Organization under a CC BY license, with permission from the World Health Organization.

Turning away from international human rights law in the 1970s, norms of equity animated WHO’s primary health care agenda under its “Health for All” strategy. This WHO strategy posited that “health is not a separate entity but an integral part of national development,” which required national and international redistributions to ensure health equity through economic development [21]. To design the contours of this socio-economic approach to horizontal public health systems, WHO convened the 1978 International Conference on Primary Health Care in Alma-Ata, USSR (now Almaty, Kazakhstan) (Fig 3), wherein states adopted the Declaration on Primary Health Care (Declaration of Alma-Ata) [22].

WHO again came to embrace human rights law in the 1980s through its Global Programme on AIDS, which recognized the importance of human rights protections for public health promotion in the early years of the global HIV/AIDS response [23]. Even as HIV/AIDS governance shifted to UNAIDS, WHO’s engagement with rights-based approaches to health would expand in the 1990s, as the UN sought to “mainstream” human rights as a cross-cutting approach to all its activities and programs [24]. Yet, WHO’s engagement with human rights advanced unevenly across WHO policies and programs [25]. While the WHO Secretariat established a Gender Advisory Panel in the 1990s to review all aspects of reproductive health with attention to gender and rights, the Organization’s focus on race and ethnicity-based discrimination would take far longer to develop. WHO framed its anti-discrimination strategy not under binding human rights law, but rather in line with norms of social justice and health equity [26].

WHO has come to merge its focus on “gender, equity and human rights”–mainstreaming these “core values” together across its work to advance justice in health. By bringing together these normative frameworks, WHO has sought to implement human rights without reference to legally binding obligations [27]–as WHO’s technical staff are seen to be resistant to legal discourses in technical programming [28]. WHO has continued to advocate for the right to health as a political foundation for the advancement of universal health coverage, developing new collaborations with the Office of the UN High Commissioner for Human Rights [29]. However, with limited WHO staff support for human rights law, human rights have often been relegated to vague principles and preambular values, limiting their transformative potential in health diplomacy [30].

## Diplomatic negotiations to develop global health law

Global health law is developed through diplomatic negotiations, primarily among sovereign states but now also including civil society and other non-state actors. Global health diplomacy—the global negotiation, adoption, implementation, and review of legal instruments, programs, and policies—has thus become central to the development of global health law under WHO governance [31]. The World Health Assembly is a principal forum for global health diplomacy. In the very first Assembly meeting in 1948, state delegates successfully adopted “Regulations No. 1 Regarding Nomenclature (Including the Compilation and Publication of Statistics) with Respect to Diseases and Causes of Death” under Articles 21 and 22 of the WHO Constitution, recognizing the importance of WHO regulations to standardize disease reporting across nations and laying out requirements for state collection and publication of causes of death [32]. The diplomatic momentum fostered by these “Nomenclature Regulations” was extended shortly thereafter by the 1951 adoption of the International Sanitary Regulations.

However, this diplomatic momentum to negotiate global health law was lost amid nearly fifty years of WHO neglect of its normative authorities. Between the early 1950s and the turn of the century, WHO was reluctant to exercise its normative function, instead revealing a preference for “technical products” to address specific diseases [33]. WHO’s landmark achievement–the eradication of smallpox–was achieved through international cooperation without legal regulation [34]. While the WHO Secretariat developed a range of non-binding tools with normative intent to influence the behavior of states, it did not formalize those activities through law [35].

**Fig 4:**
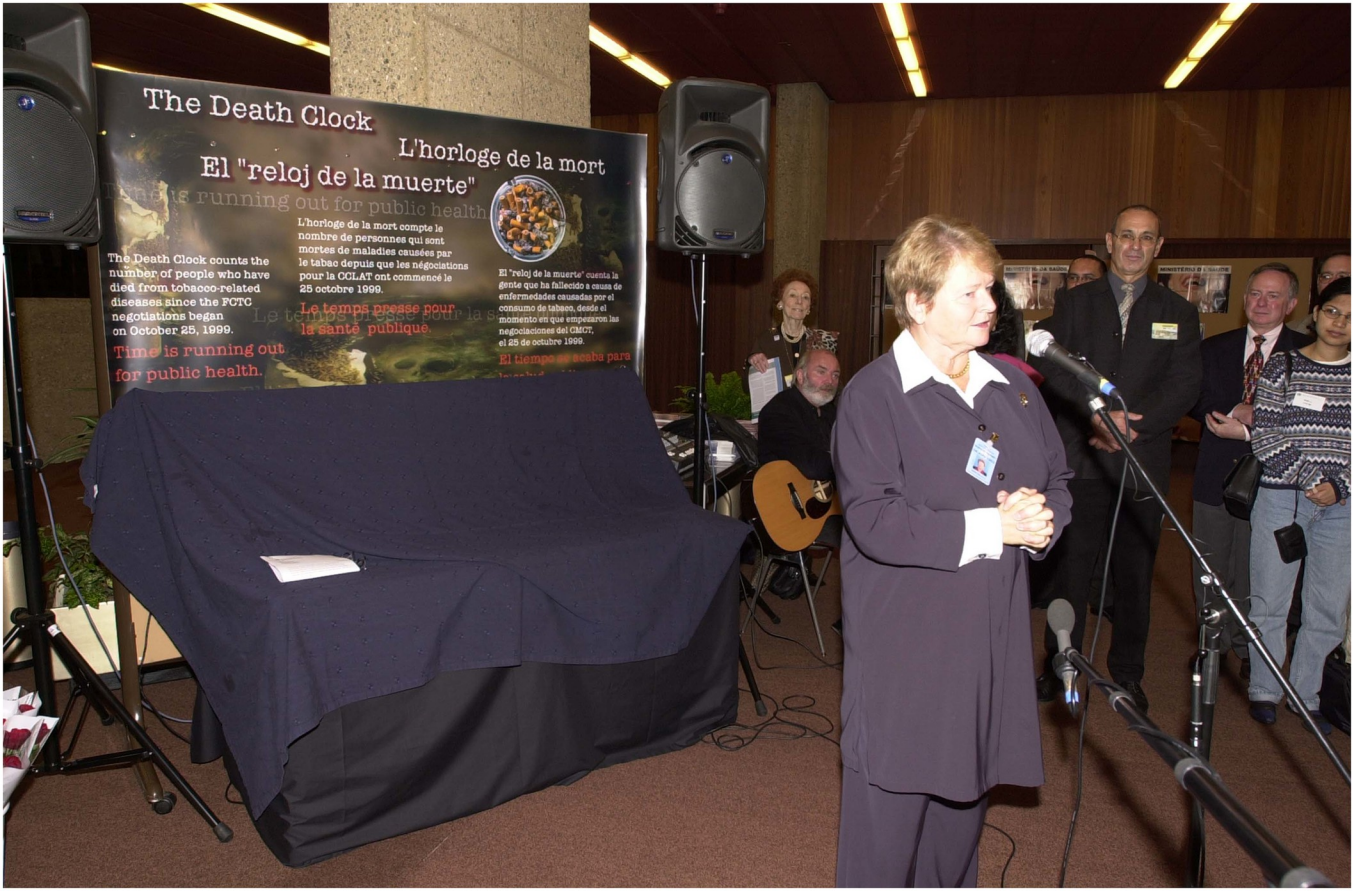
WHO director-general Gro Harlem Brundtland joining civil society in advocating for the adoption of the FCTC (Geneva, October 2002). Republished from the World Health Organization under a CC BY license, with permission from the World Health Organization.

The 21^st^ century would bring a renewed focus on global health diplomacy as a basis for the development of global health law. In advancing hard law, the negotiation and adoption of the 2003 FCTC was pivotal–for the first time ever, the World Health Assembly launched a treaty-making process under Article 19 of the WHO Constitution [36]. The FCTC negotiations saw widespread involvement from civil society, which played a crucial role in supporting a WHO treaty on tobacco, imbuing the lawmaking process with participatory legitimacy and multinational advocacy (Fig 4) [37].

The FCTC negotiations were followed rapidly by the revision of the IHR, the primary WHO instrument addressing the international spread of disease. The IHR (2005) were the result of a decade of diplomatic efforts, with negotiations catalyzed by the 2002–2003 SARS epidemic. Drawing from regional consultations, an Intergovernmental Working Group of WHO member states met in late 2004 to negotiate the final draft, which was adopted the following year by the World Health Assembly [38]. The unanimous adoption of IHR (2005) marked a renaissance for global health lawmaking under WHO–bringing the world together to address common health threats through global health diplomacy [39].

Yet despite the adoption of these hard law instruments, WHO has continued to pursue global health diplomacy through soft law advancements, seeing soft law instruments as faster and easier to adopt than international treaties, with their non-binding nature encouraging state and non-state actors to accept them more readily [40]. While lacking binding legal obligations, these soft law instruments can sometimes serve as precursors to future treaties—as demonstrated in the evolution of global tobacco regulation—overcoming diverging state interests that could lead to a political impasse [41]. Such soft law negotiations have become necessary in advancing global health governance beyond WHO–and across international institutions [42]. For example, advancing the “One Health” agenda has required close coordination with the World Organization for Animal Health (WOAH), the Food and Agriculture Organization of the United Nations (FAO), and the United Nations Environmental Programme (UNEP) [43]. Supporting sustainable development for global health has necessitated collaboration with the International Monetary Fund, the World Bank, and other economic governance actors [44]. WHO has looked to develop partnerships with diverse institutions under soft law commitments, but it will need far stronger multi-stakeholder diplomacy to respond to contemporary global health challenges, requiring multisectoral law reforms to address future pandemic challenges.

## Pandemic challenges galvanize needed reforms

The COVID-19 pandemic has tested the normative foundations of global health governance, dividing the world amid a global health threat and exposing the fragility of the international legal order. These challenges were foreshadowed by limitations of global health law in earlier Ebola responses, leading to the establishment of new multilateral policy initiatives to advance global health security–within and beyond WHO governance [45]. While WHO was intended to lead global coordination in public health emergencies, unilateral national measures violated IHR obligations and undermined WHO governance in the COVID-19 response, galvanizing necessary global health law reforms.

**Fig 5:**
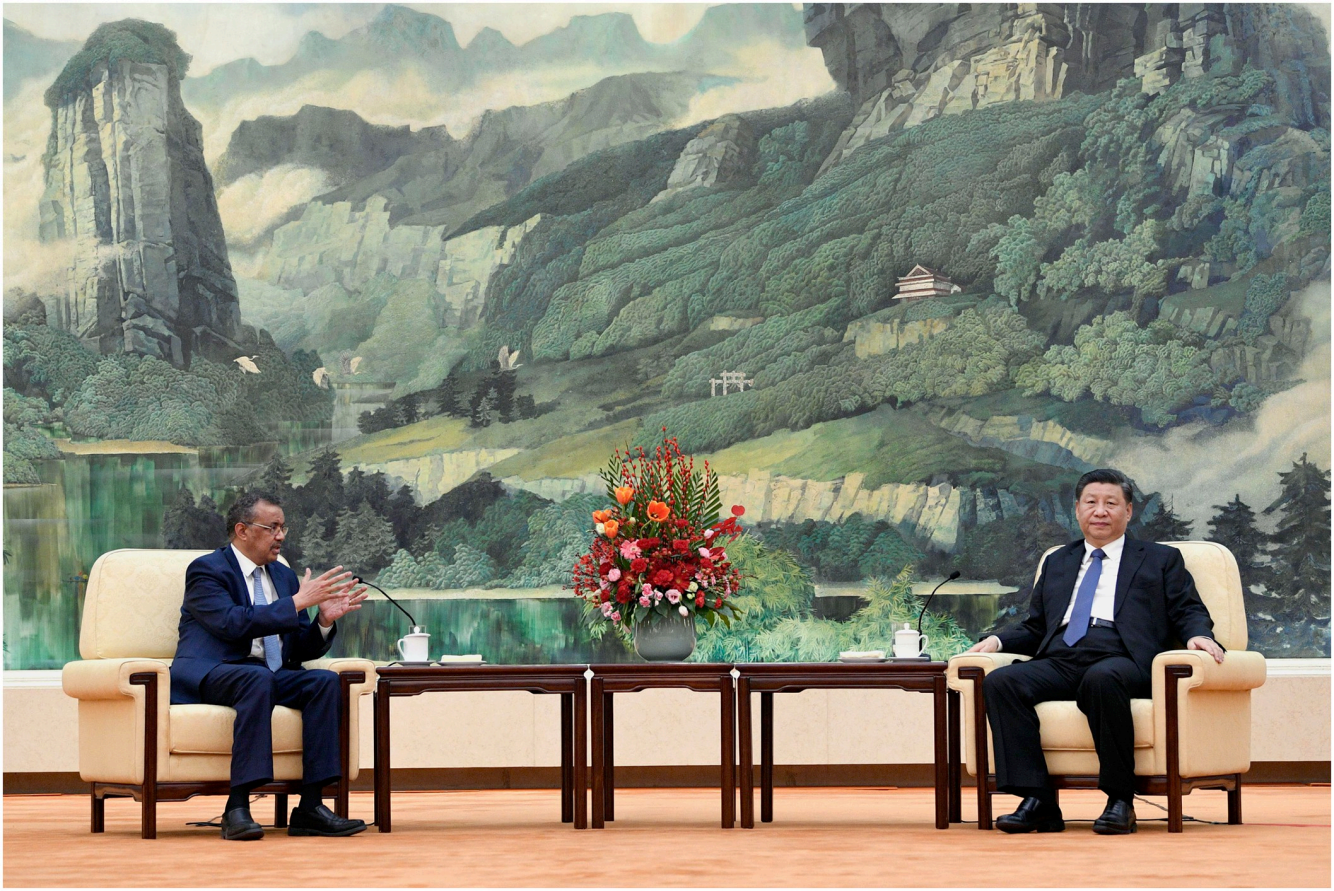
WHO director-general Tedros Adhanom Ghebreyesus meets with Chinese president Xi Jinping (Beijing, January 2020). Republished from the Associated Press under a CC BY license, with permission from the Associated Press.

The COVID-19 pandemic exposed limitations of WHO governance under the IHR (2005) [46], as seen in:

1. *Reporting public health risks to WHO*–Although the IHR require transparency and rapid notification about public health risks, China delayed reporting to WHO, repressed warnings from non-governmental sources, and withdrew information in global databases, drawing international criticism (Fig 5) [47].
2. *Coordinating national responses commensurate with public health risks*–Once WHO declared a Public Health Emergency of International Concern (PHEIC), numerous national governments neglected WHO guidance, infringed human rights, and imposed travel bans, fracturing an interconnected world [48].
3. *Supporting global solidarity in a common response*–Despite WHO efforts to rally global solidarity in the pandemic response, states neglected the long unrealized promise of international collaboration to build public health capacities and resisted new voluntary commitments to support coordination mechanisms and partnerships such as the Access to COVID-19 Tools (ACT) Accelerator [49].

WHO has continued to face challenges in efforts to ensure vaccine equity, with the pandemic response yielding to “vaccine nationalism” over vaccine supply, technology, and intellectual property, as states violated human rights obligations to realize equitable access to medical resources [50]. Notwithstanding strong statements from WHO that COVID-19 was a public health emergency and access to vaccines was a human rights imperative, many high-income states failed to participate in these solidaristic measures, and their continuing defense of intellectual property rights through the World Trade Organization (WTO) remains at odds with international obligations under WHO governance [51]. Efforts to limit waivers of the WTO Trade-Related Aspects of Intellectual Property Rights (TRIPS) and other legislative infrastructure maintaining the proprietary nature of these technologies have been barriers to the development of local manufacturing capacity–discouraging states with existing capacity from utilizing flexibilities under TRIPS to produce needed vaccines [52]. These compounding injustices in the COVID-19 response have led WHO member states to launch intergovernmental negotiations to reform global health law, laying a new foundation for binding international law in global health governance.

States are now pursuing twin law reforms to strengthen WHO governance—through both amendments to the IHR and the development of a novel pandemic agreement—with these reforms reinforcing each other to shape efforts to prevent, prepare for, and respond to future pandemics [53]. Where past reform efforts have faced challenges from WHO member states, these ongoing law reforms, taking place amid the COVID-19 pandemic, reveal renewed recognition of the need for binding legal mechanisms to foster global collaboration and preparedness for future public health emergencies.

WHO member states have proposed hundreds of amendments to the IHR that would substantially expand their scope and functions. States in the Working Group on the IHR are currently negotiating key provisions [54], including prompt and transparent reporting, scientific data sharing (including pathogen samples and genomic sequencing data), support for low- and middle-income countries, equitable access to medical countermeasures, and evidence-informed and rights-based public health measures [55]. If the World Health Assembly adopts these IHR amendments under Article 21, they would automatically enter into force for all WHO member states unless a state explicitly rejects the amendments or submits a reservation [56].

Yet, as the IHR obligations focus on outbreak detection and response rather than underlying disease prevention systems and medical countermeasures, [57] states have called for the negotiation of a separate pandemic agreement [58].

Thus, alongside the IHR amendments, states have called for a separate “convention, agreement, or other international instrument”–in what has become known as the Pandemic Accord, Pandemic Treaty, Pandemic Agreement, or CA+. This pandemic agreement would complement, rather than replace, the IHR, detailing new obligations to address limitations in global health governance and advance legal authorities that are more effective, equitable, and enforceable [59]. In 2022, an Intergovernmental Negotiating Body [60] convened by the World Health Assembly determined that this new global health law instrument will be negotiated as a convention under Article 19, with both legally binding and non-legally binding provisions, drawing on the FCTC model to become the second treaty developed under WHO [61].

Although harmonizing norms across an amended IHR and a pandemic agreement has proven challenging, these simultaneous reform processes present groundbreaking opportunities to renew global health law in pandemic prevention, preparedness, response, and recovery. The World Health Assembly has recognized the narrow window for its diplomatic efforts and seeks to conclude these interconnected negotiations in the coming months. As the most sweeping global health law developments in the past 75 years, these reforms must learn from past legal obstacles in providing for coordinated obligations across the global health governance landscape, legally-binding norms of human rights and health equity, and diplomatic negotiations that facilitate the meaningful inclusion of civil society–ensuring that necessary reforms strengthen WHO governance [62].

## Necessary reforms: Global health law to strengthen WHO governance

These legal instruments have focused global attention on WHO leadership in health emergencies, but additional reforms will be necessary to strengthen WHO governance and provide legal authorities to meet its constitutional mandate.

### Translating technical support into normative instruments

While WHO has historically presented itself as a technical organization, contemporary challenges demand normative authorities. The world is facing pressing health threats that could best be addressed through binding norms, including the looming threat of antimicrobial resistance, the backlash against sexual and reproductive health, and the health impacts of climate change [63]. WHO has vast technical expertise on these matters, but this expertise must be translated into normative guidance for member states, international organizations, civil society, and other partners. Decades of public health research preceded major legal breakthroughs like the FCTC and the International Code of Marketing of Breastmilk Substitutes, but in the absence of normative instruments, initial research often led only to incremental recommendations [64]. With states neglecting WHO recommendations, as seen in temporary recommendations in the COVID-19 response, the Organization now has an opportunity to exercise its normative function–to set global standards and facilitate accountability for state compliance.

### Mainstreaming equity and rights

WHO governance must center human rights obligations in global health law, advancing equity under global health policy and harmonizing human rights law and global health law to realize the right to the highest attainable standard of health. Overcoming human rights limitations in the COVID-19 response [65], a pandemic agreement rooted in human rights would detail obligations under the right to health to prepare for future pandemics (within and beyond the health sector), clarify human rights limitations in a public health emergency (to ensure that any rights infringements are necessary and proportional), and obligate states extraterritorially to provide international assistance and cooperation (to facilitate vaccine equity and global solidarity) [66]. With new tools to frame national health equity programs of action [67] and human rights in public health emergencies [68], an equity- and rights-centered global health law instrument would provide binding obligations to ensure justice in pandemic prevention, preparedness, recovery, and response.

### Sustainable and flexible financing

Beyond these legal obligations, WHO needs sustainable financing to replace the unpredictable funding streams that have characterized its history and politicized its work. Financing reforms must expand the sources and flexibility of WHO funding. Innovative reforms could include increased assessed contributions commensurate with WHO’s global mandate; replenishment conferences like those used by the Global Fund; revised purchasing policies that better provide cost effectiveness; increased program support fees; and partnerships for concessionary pricing [69]. In 2022, the World Health Assembly took a promising initial step toward sustainable financing by resolving to rebalance the ratio of assessed to voluntary contributions by 2030, and in 2024, the Executive Board agreed to plan for an “investment round” to ensure WHO’s base budget in line with its General Program of Work. These decisions begin to provide WHO with sustainable resources necessary to focus on the priorities decided collectively by the Assembly–rather than those of individual donors.

### A “One Health” strategy

Expanding WHO’s global health mandate, the health paradigm at the center of WHO’s role must evolve beyond the health of humans. “One Health” approaches to pandemic prevention, preparedness, and response recognize that human health is interconnected with the health of animals and our shared environment. WHO has been a crucial partner in the “Quadripartite”–an inter-organizational alliance among WHO, WOAH, FAO, and UNEP–to issue joint action plans and technical guidance on One Health. Yet, this collaboration across sectors must be strengthened under law. The proposed inclusion of One Health in global health law reforms can provide stronger normative grounding for WHO efforts at the center of this multi-sectoral and inter-organizational approach to global health law–including stricter regulation and oversight of illicit wildlife trade, industrial agriculture, and environmental threats [70].

### Good governance

Good governance seeks to apply principles of accountability, transparency, participation, solidarity, and equity to global health governance. WHO has made notable strides toward transparency and participation in policymaking, most notably through the 2016 adoption of its Framework of Engagement with Non-State Actors (FENSA). However, FENSA has been widely criticized, with scholars and advocates critiquing the ways in which civil society organizations and multinational corporations are treated in the same way [71]. Where private sector influence has undermined trust in the Organization at difficult times, further reforms will be necessary to ensure inclusive participation, including more proactive public disclosure of participants in important meetings and committees, terms of agreements with private sector partners, and commitments to transparency in internal operations. Such governance reforms can preserve trust in WHO leadership, with meaningful civil society engagement informing WHO decision-making and elevating public support.

Drawing from past struggles, these reforms will require efforts to revitalize hard law authorities in global health, strengthen WHO leadership across the global governance landscape, uphold equity and rights at the center of global health law, and expand negotiations in global health diplomacy.

## Conclusion: Ensuring global governance under global health law

WHO has confronted unprecedented challenges over the past seventy-five years, and it will be necessary to strengthen global health governance to face future challenges through global health law–looking to past successes and missed opportunities in deploying hard law authorities, coordinating multisectoral actors, mainstreaming human rights norms, and engaging in inclusive diplomacy. Reforms are urgently needed to realize the promise of WHO’s normative function, but as WHO prepares for future global health challenges, member states find themselves at a crossroads in the development of global health law–either to accept the divisive responses that have characterized the response to COVID-19 or to recommit to international cooperation through WHO governance. The next generation of global health governance will be born from the coming reforms of global health law.
